# CQMUH-011 Inhibits LPS-Induced Microglia Activation and Ameliorates Brain Ischemic Injury in Mice

**DOI:** 10.1007/s10753-021-01420-3

**Published:** 2021-02-02

**Authors:** Hailin Liu, Xiangnan Hu, Rong Jiang, Jianghui Cai, Qiao Lin, Zhiguo Fan, Pan Zhao, Song Wang, Chunqiao Zou, Weimin Du, Zhi Dong, Yingju Liu

**Affiliations:** 1 Department of Pharmacology, the Key Laboratory of Biochemistry and Molecular Pharmacology, Chongqing Medical University, Chongqing, 400016 China; 2 Department of Pharmacy, First People’s Hospital of Chongqing Liangjiang New zone, Chongqing, 401121 China; 3 College of Basic Medicine, Chongqing Medical University, Chongqing, 400016 China

**Keywords:** CQMUH-011, microglia, neuroinflammation, NF-κB, MAPKs, cerebral ischemia/reperfusion injure

## Abstract

Excessive microglial activation-mediated neuroinflammation is closely involved in the pathogenesis of several neurological diseases. CQMUH-011, as a novel adamantane sulfonamide compound, has been shown anti-inflammatory properties in activated macrophages (RAW264.7). However, the role of CQMUH-011 in microglial activation-induced neuroinflammation and neuroprotective properties has yet to be elucidated. In the present study, we investigated the potential effects and mechanisms of CQMUH-011 on lipopolysaccharide (LPS)-stimulated primary microglia *in vitro* and transient middle cerebral artery occlusion (t-MCAO)–induced acute cerebral ischemia/reperfusion (I/R) injury *in vivo*. The results demonstrated that CQMUH-011 significantly suppressed the production of tumor necrosis factor (TNF)-α and interleukin (IL)-1β by LPS-stimulated primary microglia. In addition, CQMUH-011 inhibited the proliferation of activated microglia by arresting the cell cycle at the G_1_/S phase accompanied by downregulating the expression of cell cycle regulatory proteins such as proliferating cell nuclear antigen (PCNA) and cyclin D1. CQMUH-011 was seen to induce apoptosis in activated microglia by regulating the expression of Bax and Bcl-2. Furthermore, CQMUH-011 markedly attenuated the protein expression of Toll-like receptor 4 (TLR4) and myeloid differentiation factor 88 (MyD88) as well as the phosphorylation levels of nuclear factor-kappa (NF-κB) subunit p65, inhibitory kappa B-alpha (IκBα), and mitogen-activated protein kinases (MAPKs) such as extracellular signal-regulated kinase (ERK) and p38 kinases. *In vivo*, CQMUH-011 administration significantly improved neurological function and infarct volume, and ameliorated the inflammatory cytokines and microglia amount around the injury site of mice. In conclusion, these results suggested that CQMUH-011 has a notable anti-inflammatory effect and protects mice from I/R injure. Thus, CQMUH-011 may be a candidate drug for the treatment of cerebral ischemia patients.

## INTRODUCTION

Neuroinflammation exerts a crucial role in neurological disease and has been under intense investigation in recent years [1, 2]. Microglia, the resident phagocytic cells of the central nervous system (CNS), are the main effectors of this neuroinflammation. Microglial activation-mediated neuroinflammation is closely associated with various neurological disorders [3, 4]. Therefore, neuroinflammation has been a new therapeutic target not only in acute brain disorders such as acute ischemic stroke [5] but also in chronic degenerative neurological diseases such as Parkinson’s disease, Alzheimer’s disease (AD), and multiple sclerosis [6, 7].

As the major innate immune cells in the CNS, microglia have been shown to play dual roles in response to abnormal stimulation such as ischemic stroke. Microglia exhibit various behaviors in pathological conditions, including division, activation, and transference to the injury site [3, 8]. On the one hand, activated microglia promote neurogenesis by secreting various neurotrophic factors and removing cytotoxic metabolites to stabilize the microenvironment for neurons, which typically prevent pathophysiological processes. On the other hand, excessive microglial activation induces significant neurotoxic effects by the excess production of inflammatory cytokines and chemokines such as tumor necrosis factor (TNF)-α, inducible nitric oxide synthase (iNOS), nitric oxide (NO), and interleukin (IL)-1β [9], which may play a critical role in the pathogenesis of ischemic stroke [10]. Therefore, much attention has been paid to therapeutic strategies that suppress neurotoxic microglial activation.

CQMUH-011 (C_23_H_30_N_3_O_4_S, 457 g/mol, Patent Application No. 201610818842.7, P.R.C), a compound with a novel structure, was synthesized by our research group. Our previous study demonstrated that CQMUH-011 exhibited anti-inflammatory and protective effects on lipopolysaccharide (LPS)/d-GalN-induced fulminant hepatic failure (FHF) by downregulating the production of various pro-inflammatory cytokines and mediators, thus inhibiting the activation of macrophages [11]. However, it is unclear whether CQMUH-011 interferes with the inflammatory process in the nervous system. In the present study, we investigated the effects of CQMUH-011 on LPS-activated primary microglia and explored the possible mechanism of action of CQMUH-011 on LPS-induced neuroinflammation *in vitro*. And explored the potential effects of CQMUH-011 on transient middle cerebral artery occlusion (t-MCAO) induced acute cerebral ischemia/reperfusion (I/R) injury.

## MATERIALS AND METHODS

### Materials

CQMUH-011 (purity > 95%) was provided by the Chongqing Key Laboratory of Biochemistry and Molecular Pharmacology (Chongqing, China). Fetal bovine serum (FBS), Dulbecco’s Modified Eagle’s Medium (DMEM), and penicillin and streptomycin were obtained from GIBCO (Life Technologies, Grand Island, NY, USA). Trypsin-EDTA was purchased from Thermo Scientific (Waltham, MA, USA). TNF-α, IL-1β, and ELISA kits were purchased from Boster (Wuhan, China). LPS (*Escherichia coli*, 055:B5) and 3-(4,5-dimethylthiazol-2-yl)-2,5-diphenyltetrazolium bromide (MTT) were obtained from Sigma-Aldrich (St. Louis, USA). Antibodies against extracellular signal-regulated kinase (ERK), phospho-ERK, phospho-nuclear factor-kappa (NF-κB) p65 (P-p65), inhibitory kappa B-alpha (IκBα), phospho-IκBα, p38 mitogen-activated protein kinases (MAPK), and phospho-p38 (MAPK) were purchased from Cell Signaling Technology (Danvers, MA, USA). Antibodies against proliferating cell nuclear antigen (PCNA), Cyclin D1, Bax, Bcl-2, IBA1, OX-42, Toll-like receptor 4 (TLR4), and myeloid differentiation factor 88 (MyD88) were purchased from Santa Cruz Biotechnology, Inc. (Santa Cruz, CA, USA). Antibodies against β-actin and horseradish peroxide (HRP)–conjugated goat anti-rabbit IgG (H + L) were purchased from Proteintech Biotechnology (Proteintech, Wuhan, China).

### Primary Microglial Culture

Primary microglial cultures were purified from primary mixed glial cultures as previously described [12]. Briefly, the cerebral cortices were dissected from 1-day-old C57BL6 mice. To obtain mixed glial cells, the brain was mechanically dissociated, the meninges and blood vessels were removed, and then the tissue was digested with 0.125% trypsin-EDTA solution for 15 min at 37 °C. The isolated cells were seeded in culture flasks and maintained in DMEM/F12 containing 10% FBS, 100 U/mL penicillin, and 100 g/mL streptomycin for 2 weeks at 37 °C in a humidified atmosphere of 5% CO_2_. The medium was changed every 3 days. Microglial cells were isolated from mixed glial cultures by shaking the flasks for 1 h at 260 rpm. The medium was harvested, and after 1 h of incubation at 37 °C, the non-adherent cells were removed and plated onto new plates for subsequent experiments. The purity of microglial cells was monitored using OX-42 Ab *via* flow cytometry, and 95% of cells were stained positively.

### MTT Assay

MTT was used to evaluate the effect of CQMUH-011 on cell viability. Briefly, primary microglial cells were plated in 96-well plates at a density of 6000 cells/well and incubated overnight. The cells were treated with different concentrations of CQMUH-011 (0, 0.003, 0.01, 0.03, 0.1, 0.3, and 1 μM) for 24 h and co-cultured in the absence or presence of 100 ng/mL LPS for another 24 h. After incubation with 5 mg/mL MTT working solution for 4 h at 37 °C, the formazan crystals that formed were dissolved with 150 μL of dimethyl sulfoxide (DMSO). The absorbance at 490 nm was measured with a PR 4100 microplate reader.

### Enzyme-Linked Immunosorbent Assay

The production of pro-inflammatory cytokines (IL-1β and TNF-α) in culture medium and ischemic brain tissue homogenate were quantified using an ELISA kit according to the manufacturer’s instructions.

### Immunofluorescence Staining

Primary microglial cells and brain tissue sections were identified by immunofluorescence staining. For primary microglial cells, the coverslips were washed with phosphate-buffered saline (PBS) three times and fixed with 4% paraformaldehyde for 10 min at room temperature. After washing with PBS, the coverslips were incubated in 0.2% Triton-100 for 10 min and blocked with 5% bovine serum albumin (BSA) for 1 h. For brain tissue sections, 3 days after t-MCAO, mice from each group (*n* = 3) were deeply anesthetized and transcardially perfused with heparinized saline followed by 4% neutral-buffered paraformaldehyde. Brains were removed and fixed with the same solution for 12 h. Frozen sections of the brain, 5 μm thick. Then, the cells were incubated with anti-IBA-1 mouse monoclonal antibodies (sc-32725, Santa Cruz, CA, USA, 1:200,) and brain sections were incubated with anti-IBA-1 (48934s, Cell Signaling Technology, MA, USA, 1:200) at 4 °C overnight. Coverslips and sections were washed with PBS and incubated with FITC-conjugated goat anti-mouse IgG (A22110 and A22120, respectively, Abbkine, Redlands, CA, USA 1:200) for 2 h. Nuclei were stained with DAPI (C1006, Beyotime, Shanghai, China) for 5 min at room temperature. Images were captured with a fluorescence microscope (Eclipse Ti-S; Nikon, Tokyo, Japan).

### Cell Cycle and Apoptosis Analysis by Flow Cytometry

As described previously [11], primary microglial cells were seeded at a cell density of 1 × 10^6^ cells per mL in 6-well plates. After undergoing different treatments, the cells were digested with trypsin and washed with cold PBS three times. For the cell cycle assay, the cells were then fixed with ice-cold 70% ethanol overnight at 4 °C. Subsequently, the cells were centrifuged and resuspended in 1 mL of PBS and then stained with 1 mL of propidium iodide (PI) solution. After 1 h, the cell cycle phase distribution was analyzed by a BD Accuri C6 flow cytometer (BD, Franklin Lakes, NJ, USA) according to the manufacturer’s instructions. To explore the effect of CQMUH-011 on apoptosis, the cells were resuspended in a phosphate buffer solution, stained with fluorescein isothiocyanate-conjugated annexin V for 10 min at room temperature, centrifuged and resuspended in binding buffer mixed with PI (1 mg/mL). The apoptotic rate was detected by flow cytometry.

### Western Blotting

Microglial cell pellets were collected to separate the cytoplasmic and nuclear fractions using the nuclear extraction kit (Abcam, #ab113474, Cambridge, MA, USA) according to the manufacturer’s instructions. Cells were collected in RAPI lysis buffer (P0013D, Beyotime, Shanghai, China). The protein concentrations of the samples were determined using a BCA protein assay kit (P0012S, Beyotime, China). An 8-μL sample of the protein was separated by sodium dodecyl sulfate polyacrylamide gel electrophoresis (SDS-PAGE) and transferred to a polyvinylidene fluoride (PVDF) membrane (Millipore, USA). The membranes were blocked for 1 h with 5% BSA at room temperature and then incubated with specific primary antibodies: rabbit polyclonal anti-ERK(1/2) (#9102, Cell Signaling Technology, MA, USA, 1:1000), rabbit polyclonal anti-phospho-ERK (1/2) (#4376, Cell Signaling Technology, MA, USA, 1:1000), mouse monoclonal anti -Phospho-NF-κB p65 (#13346, Cell Signaling Technology, MA, USA, 1:1000), mouse monoclonal anti-IκBα (#9247, Cell Signaling Technology, MA, USA, 1:1000), mouse monoclonal anti-phospho-IκBα (#9246, Cell Signaling Technology, MA, USA, 1:1000), rabbit polyclonal anti-p38 (#9212, Cell Signaling Technology, MA, USA, 1:1000), rabbit polyclonal anti-Phospho-p38 (#9211, Cell Signaling Technology, MA, USA, 1:1000), mouse monoclonal anti-PCNA(sc-53407, Santa Cruz, CA, USA, 1:1000), rabbit polyclonal anti-cyclin D1 (sc-717, Santa Cruz, CA, USA, 1:1000), mouse monoclonal anti-Bax (sc-70407, Santa Cruz, CA, USA, 1:1000), mouse monoclonal anti-Bcl-2 (sc-23960, Santa Cruz, CA, USA, 1:1000), rabbit polyclonal anti-TLR4 (sc-10741, Santa Cruz, CA, USA, 1:1000), rabbit polyclonal anti-MyD88 (sc-11356, Santa Cruz, CA, USA, 1:1000), and rabbit polyclonal anti-β-actin (20536-1-AP, Proteintech, Wuhan, China, 1:3000). The membranes were washed three times in Tris buffered saline with Tween (TBST) and incubated with HRP-conjugated secondary antibodies for 1 h at room temperature. Following three washes in TBST, immunoreactive bands were observed using an enhanced chemiluminescence detection system (Bio-Rad, Hercules, CA, USA).

### Animals

Adult male C57BL/6 mice, weighing 20–25 g, were obtained from the Animal Laboratory Center of Chongqing Medical University (Chongqing, China). All animals were housed in a 12-h light-dark cycle *ad libitum* with temperature-controlled (25 ± 1 °C) facility and maintained on standard food and water available. All animal experimental protocols were in accordance with the guidelines of the National Institutes of Health and were approved by the Experimental Ethics Committee of Chongqing Medical University (License number: SYXK YU 2010-001).

The cerebral ischemia-reperfusion model was induced by transient middle cerebral artery occlusion (t-MCAO) lasting for 60 min, as our previously described [13]. Forty-eight male C57BL/6 mice were randomly separated into the following experimental groups: (1) sham group, (2) I/R (vehicle-operated) group, (3) CQMUH-O11-treated I/R group. The solution of CQMUH-O11 was prepared as previously described [11]. The CQMUH-011(75 μg/kg, 225 μg/kg) or the vehicle was administered by intraperitoneal injection immediately after t-MCAO (60 min), and the injections were repeated daily injection until were sacrificed.

### Behavioral Testing

The neurologic deficit evaluation was performed before t-MCAO and 1, 3, 7, and 14 days after the procedure. The test was performed by two investigators who were blinded to the experimental. The result of the mNSS test is a composite of general, focal, balance, reflex, and sensory tests with a minimum of 0 point in normal mice to a maximum of 18 points in severely injured mice [13, 14].

### Measurement of Infarction Volume

Infarct volume was quantified by 2,3,7-triphenyltetrazolium chloride (TTC) staining as described previously [13, 15]. Briefly, 24 h after t-MCAO, the brains were quickly isolated, frozen, and sliced into 2-mm thick consecutive coronal slices. The slices were immersed into 2% TTC solution for 15 min at 37 °C and fixed with 4% paraformaldehyde at 4 °C overnight. Images of the slices were captured by a digital scanner, and the infarct volume was calculated using Image-Pro Plus6software (Media Cybernetics, Rockville, MD, USA).

### Statistical Analysis

All results are expressed as the means ± standard deviation (SD), and SPSS 17.0 software was used to perform the statistical analyses. Comparisons among different groups were performed with one-way analysis of variance (ANOVA) followed by Tukey’s test. *P* values < 0.05 were considered statistically significant.

## RESULTS

### Effect of CQMUH-011 on Microglial Cell Viability and Toxicity

**Fig. 1:**
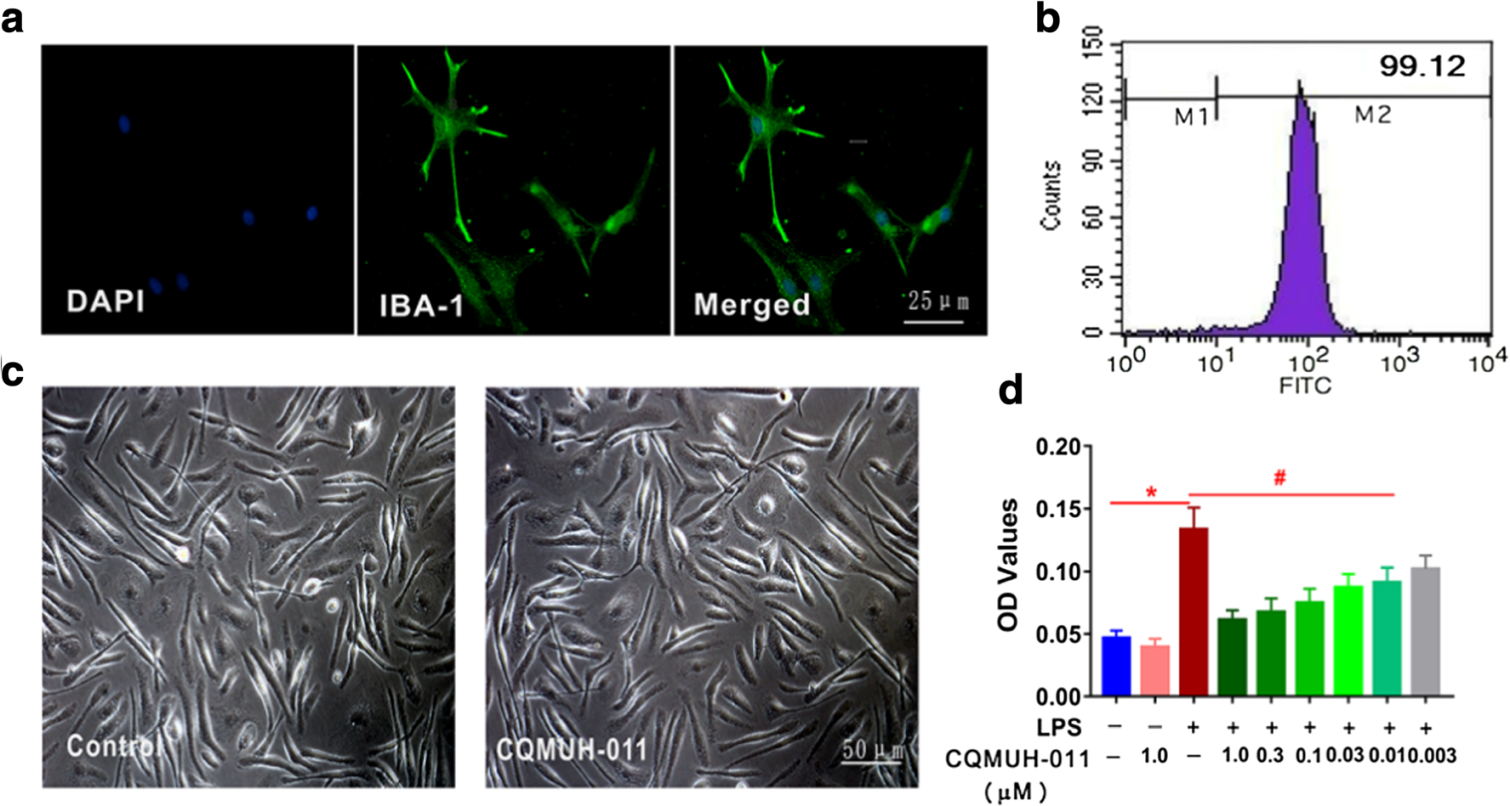
Effect of CQMUH-011 on microglial viability. **a** Identification of primary microglia by immunofluorescence staining for IBA-1 (scale bar = 25 μm). **b** The purity of the microglial culture was calculated by counting OX-42-positive cells based on flow cytometry. **c** Representative morphologies of microglia after CQMUH-011 treatment (1 μM) for 24 h (scale bar = 50 μm). **d** MTT assay of microglial cell viability after CQMUH-011 treatment. Data shown are the mean ± SD (*n* = 3 independent experiments), *P < 0.05 LPS group *vs.* control group; ^#^*P* < 0.05 CQMUH-011 group *vs.* LPS group.

As shown in Fig. 1a, the primary microglial cells were detected with specific anti-IBA-1 antibodies *via* an immunofluorescence assay. Then, we investigated the purified primary microglial cells by flow cytometry. As shown in Fig. 1b, more than 99.12% of primary cells were positive for OX-42, which is a known marker of microglia [16]. To further identify cytotoxicity, microglia were exposed to CQMUH-011 (1 μM) for 48 h. No obvious morphological changes were observed in the treated microglia compared with the control group (Fig. 1c). The potential effect of CQMUH-011 on microglial proliferation and cell toxicity was evaluated using the MTT cell viability assay. As shown in Fig. 1d, the viability of primary microglia was remarkably increased by LPS treatment compared with that of the control group, while pretreatment with various concentrations of CQMUH-011 (0.01, 0.03, 0.1, 0.3, and 1 μM) showed an inhibitory effect on microglial proliferation in a dose-dependent manner (*P* < 0.05). The IC_50_ value (half of the maximal inhibitory concentration) was found to be 1.097 × 10^−8^ mol·L^−1^ with a 95% confidence interval ranging from 0.8754 × 10^−8^ mol·L^−1^ to 1.374 × 10^−8^ mol·L^−1^. These results suggest that CQMUH-011 has no cytotoxicity to microglia in a normal condition but significantly inhibits the proliferation of microglia stimulated by LPS.

### CQMUH-011 Inhibits the Release of Pro-inflammatory Cytokines

**Fig. 2:**
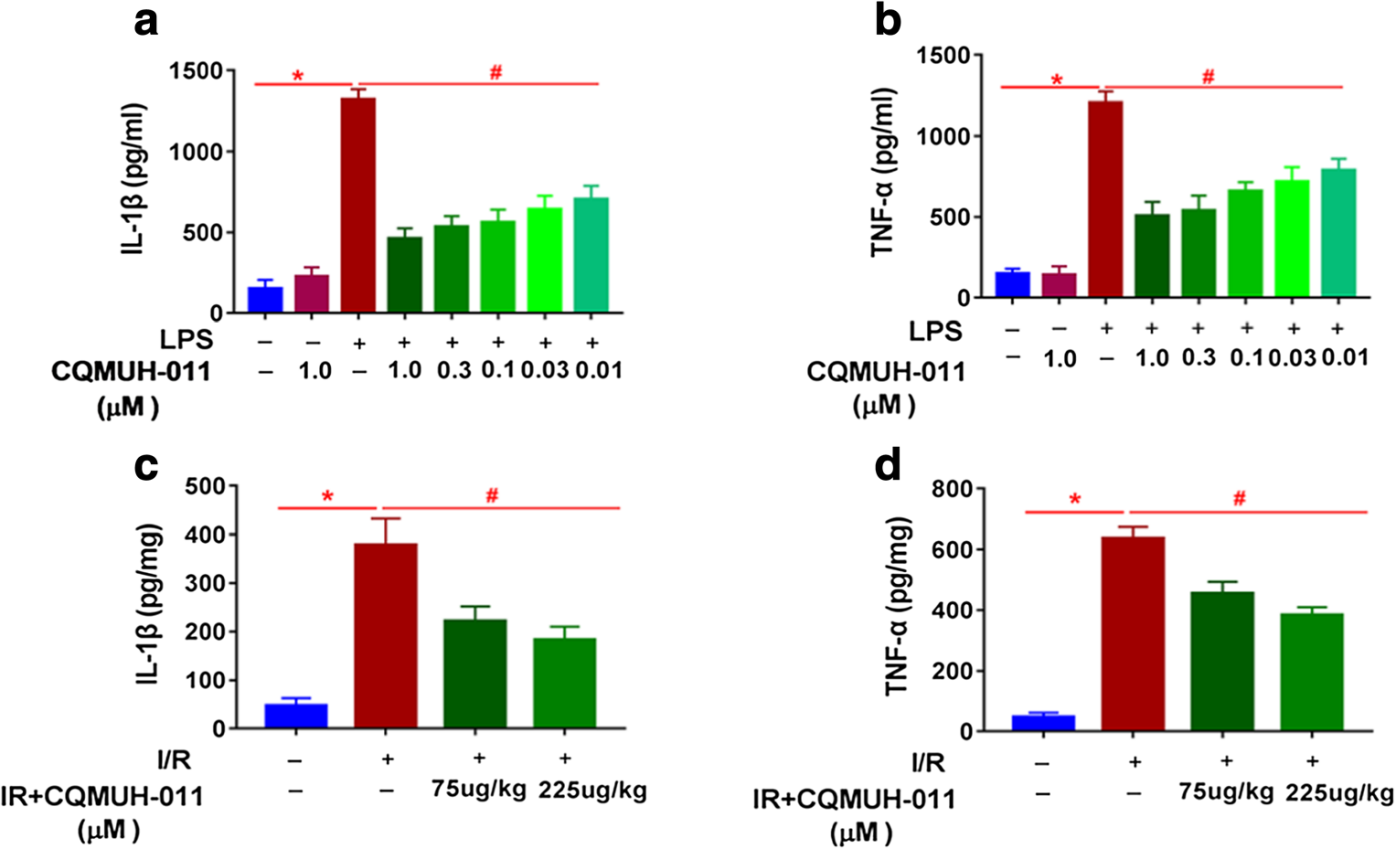
Inhibitory effects of CQMUH-011 on IL-1β and TNF-α production *in vitro* and *in vivo*. *In vitro*, primary microglial cells were pretreated with CQMUH-011 for 24 h prior to incubation with LPS (100 ng/mL) for another 24 h. *In vivo*, 3 days after t-MCAO, the brain tissue around the injury site was orderly harvested and washed. The homogenate was centrifuged at 5000*g* for 5 min. Levels of cytokines IL-1β and TNF-α in the supernatant were measured by ELISA. **a**, **c** The level of IL-1β. **b**, **d** The level of TNF-α. Each value is the mean ± SD (*n* = 3). *P < 0.05 LPS group *vs.* control group; ^#^*P* < 0.05 CQMUH-011 group *vs.* LPS group.

The expression of pro-inflammatory factors such as TNF-α and IL-1β were analyzed in LPS-stimulated microglial cells and cortex around injury site of mice. As shown in Fig. 2a, b, the levels of IL-1β and TNF-α were significantly higher after stimulation with LPS for 24 h compared to those in cells without treatment (*P* < 0.05). Pretreatment with various concentrations of CQMUH-011 significantly downregulated the expression levels of IL-1β and TNF-α. As shown in Fig. 2c, d, the levels of TNF-α and IL-1β secretion in the I/R group was vastly enhanced on day 3 post-t-MCAO. Compared with the I/R group, CQMUH-011 administration significantly decreased the concentration of TNF-α and IL-1β. These results demonstrated that CQMUH-011 reduced inflammatory responses both *in vitro* and *in vivo*.

### CQMUH-011 Arrests the Cell Cycle and Induces Apoptosis in LPS-Stimulated Microglia

**Fig. 3:**
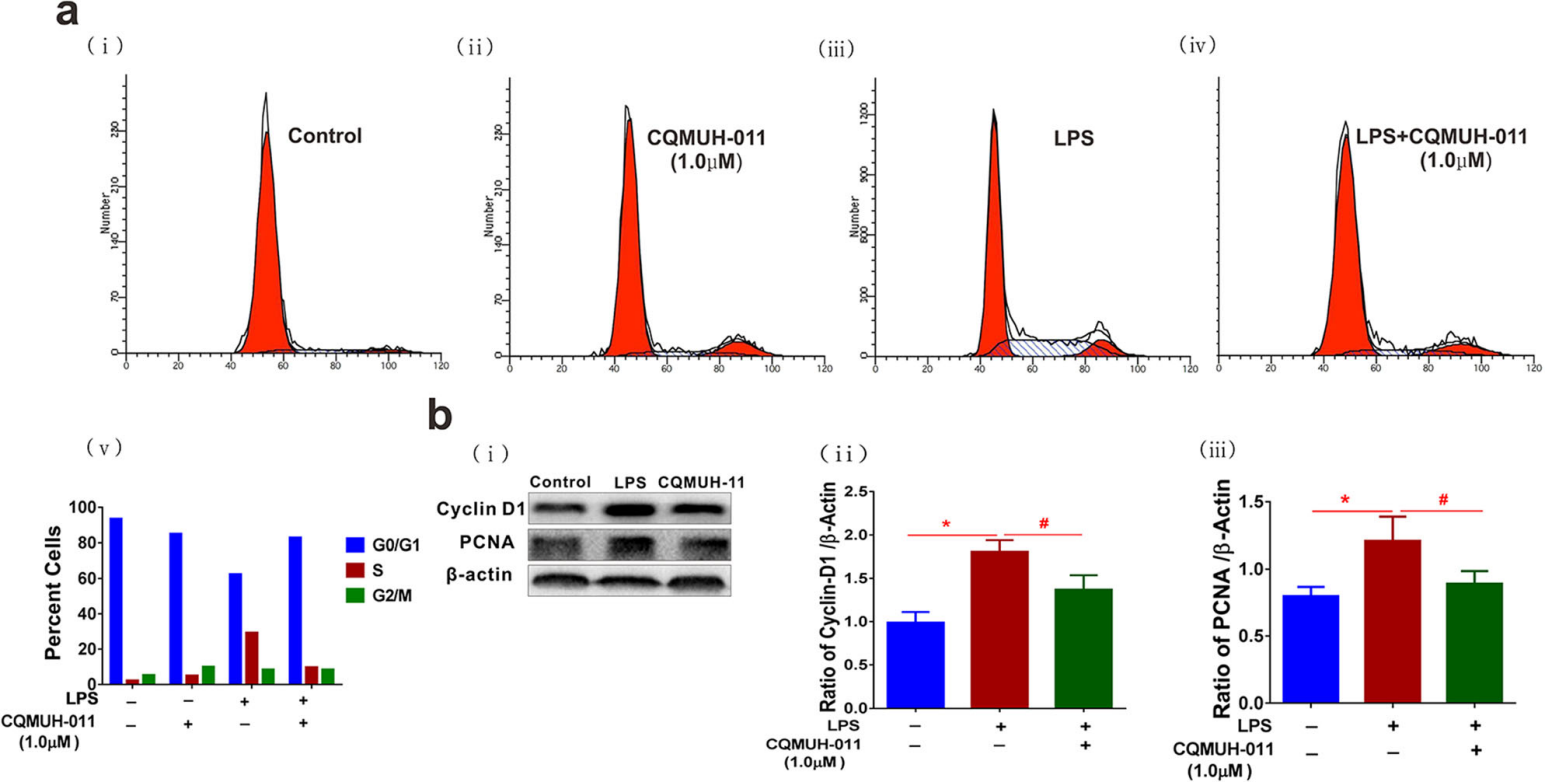
Inhibitory effects of CQMUH-011 on LPS-induced proliferation in primary microglial cells. **a** (i–iv) Representative plots showing the cell cycle analysis of the CQMUH-011-treated primary microglia with or without activation with LPS analyzed by flow cytometry, and **a** (v) histogram presenting the distribution of cells in G0/G1, S, and G2/M phases of the cell cycle in different groups. **b** (i) Western blot analysis of cyclin D1 and PCNA protein expression levels in LPS-induced primary microglial cells. **b** (ii, iii) Histograms presenting quantification of cyclin D1 and PCNA protein expression normalized to endogenous β-actin in different groups. Data are expressed as the means ± SD (*n* = 3 per group). *P < 0.05 LPS group *vs.* control group; ^#^*P* < 0.05 CQMUH-011 group *vs.* LPS group.

We investigated the effect of CQMUH-011 on the cell cycle and apoptosis in LPS-stimulated microglial cells. Flow cytometry was performed to assay the cell cycle distribution of microglia after different treatments. The results revealed that primary microglia stimulated with LPS clearly increased their proliferation rate with 62.25% cells in the G0/G1, 29.34% in the S, and 8.41% in the G2/M phases of the cell cycle compared to cells without treatment (92.55% cells in the G0/G1, 2.18% in the S, and 5.27% in the G2/M phases). However, CQMUH-011 efficiently inhibited this LPS-induced proliferation by arresting the G1/S transition (82.94% cells in the G0/G1, 9.7% in the S, and 7.36% in the G2/M phases) (Fig. 3a). Consistent with these data, CQMUH-011 markedly downregulated the expression of the cell cycle regulatory proteins PCNA and cyclin D1 (Fig. 3b), both of which play key roles in cell cycle progression [17].

**Fig. 4:**
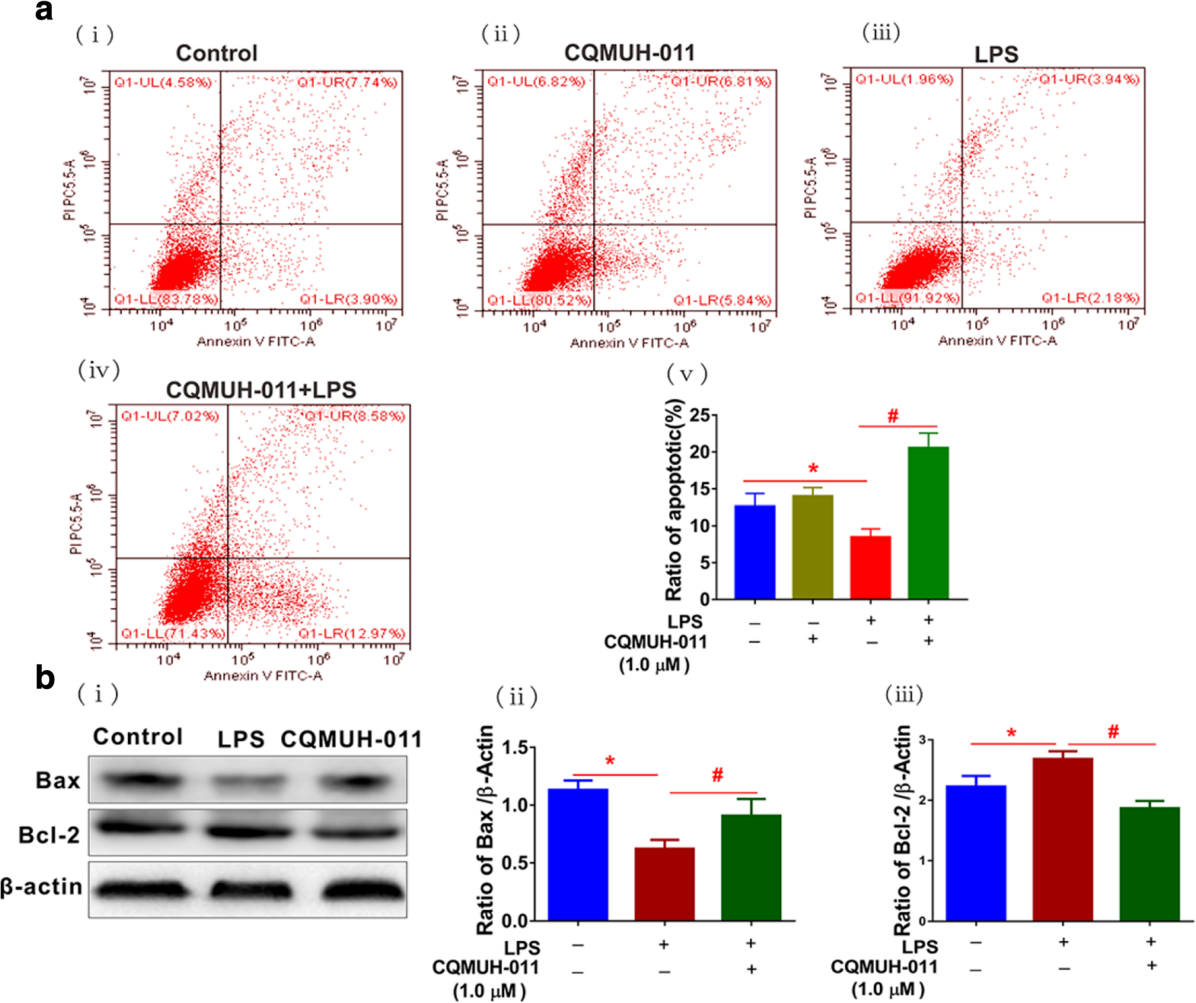
CQMUH-011 induces apoptosis in LPS-activated primary microglia. **a** (i–iv) Representative plots showing the annexin-FITC assay for CQMUH-011-treated primary microglia with or without activation with LPS by flow cytometry. **a** (v) Histogram presenting the rate of apoptosis in each group. Each value is the mean ± SD (*n* = 3). ^**#**^*P* < 0.05 *vs.* LPS group. **b** (i) Western blot analysis of Bax and Bcl-2 protein expression levels in LPS-induced primary microglial cells. **b** (ii, iii) Histograms presenting quantification of Bax and Bcl-2 protein expression normalized to endogenous β-actin in different groups. Data are expressed as the means ± SD (*n* = 3 per group). *P < 0.05 LPS group *vs.* control group. ^#^*P* < 0.05 CQMUH-011 group *vs.* LPS group.

Next, we investigated the impact of CQMUH-011 on cell apoptosis using the annexin V-FITC assay. Our results illustrated that treatment of microglia with LPS induced a significantly lower apoptosis rate compared to cells without treatment. However, pretreatment with CQMUH-011 clearly prevented the reduction of apoptotic phase cells (Fig. 4a). Moreover, LPS treatment significantly downregulated the expression of the pro-apoptotic protein Bax, whereas CQMUH-011 pretreatment significantly improved its expression and downregulated the expression of the apoptotic cascade protein Bcl-2 (Fig. 4b). These results revealed that pretreatment with CQMUH-011 arrested the cell cycle and promoted apoptosis in activated primary microglia.

### Effects of CQMUH-011 on TLR4, MyD88, and NF-κB Expression in LPS-Stimulated Microglia

**Fig. 5:**
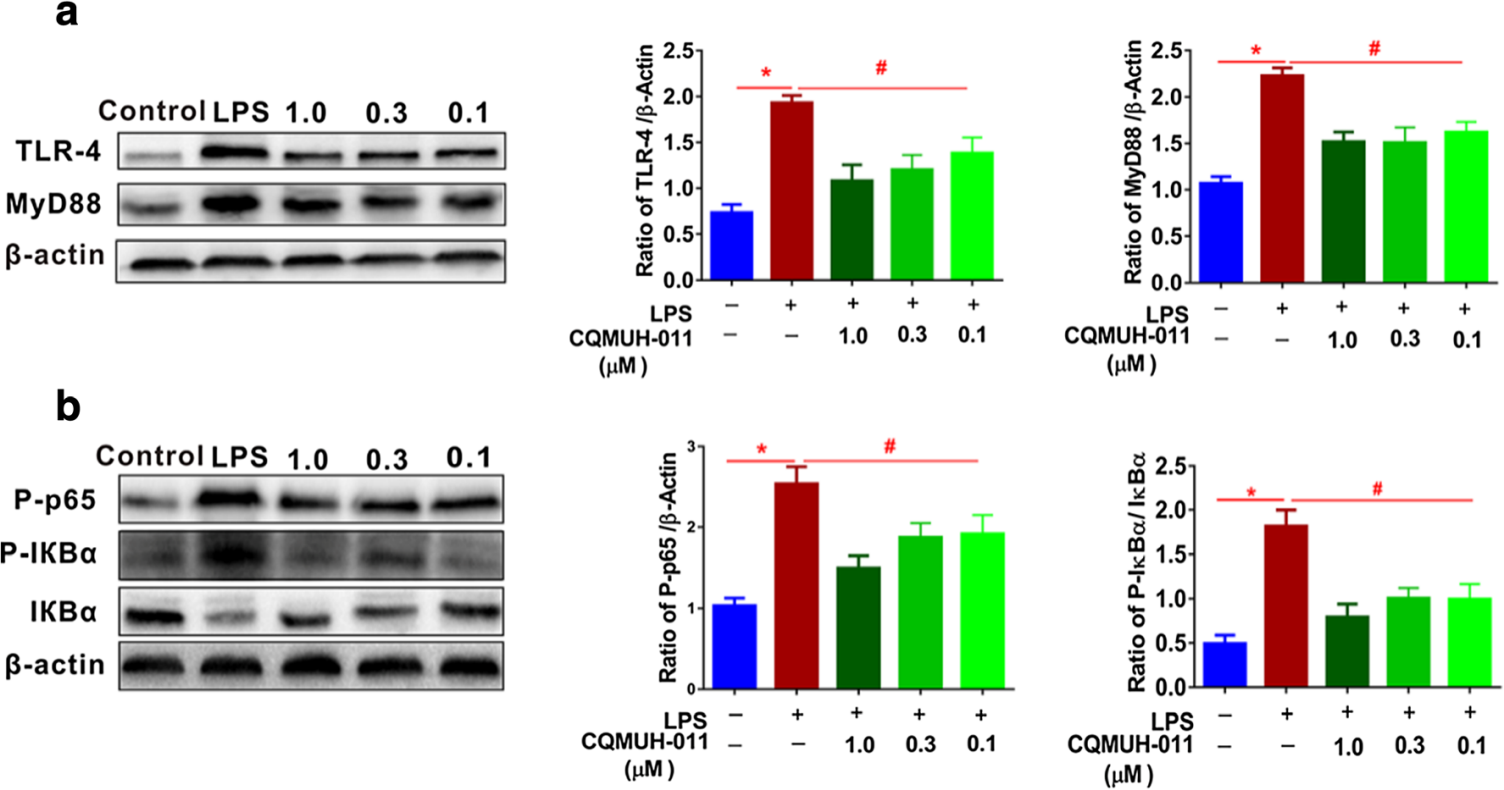
Inhibitory effects of CQMUH-011 on TLR4-MyD88 and NF-κB signaling. **a** Western blot analysis of TLR4 and MyD88 expression in LPS-induced primary microglial cells. Histograms presenting quantification of TLR4 and MyD88 expression normalized to endogenous β-actin in different groups. **b** Western blot analysis of P-p65, P-IκBα, and IκBα expression in LPS-induced primary microglial cells. Histograms presenting quantification of P-p65 expression normalized to endogenous β-actin and relative ratios of phosphorylated to total IκBα in different groups. Data are expressed as the means ± SD (*n* = 3 per group). *P < 0.05 LPS group *vs.* control group; ^#^*P* < 0.05 CQMUH-011 group *vs.* LPS group.

It is well-known that TLR4 is highly specific in its ability to recognize LPS, and it plays an important role in regulating the expression of inflammatory mediators through the activation of NF-κB. Therefore, the effects of CQMUH-011 on LPS-induced TLR4 expression were examined. As shown in Fig. 5a, the expression of TLR4 in LPS-stimulated microglial cells was significantly higher than that in cells without treatment. However, pretreatment with CQMUH-011 clearly decreased the expression of TLR4 in a dose-dependent manner. Myeloid differentiation factor 88 (MyD88) acts as an adapter and is involved in TLR4 signal transduction and NF-κB activation [18]. Therefore, the effects of CQMUH-011 on MyD88 were also detected. As shown in Fig. 5a, after the treatment of primary microglial cells with LPS, MyD88 protein levels markedly increased but were suppressed upon treatment with CQMUH-011. These data suggest that TLR4 and MyD88 proteins are involved in the anti-neuroinflammatory effects of CQMUH-011 on LPS-stimulated primary microglial cells.

It is well-established that the NF-κB signaling pathway is closely associated with the inflammatory response. Under normal physiological conditions, IκBα, an inhibitory protein, functions as a modulator to sustain the deactivation of NF-κB. Various extracellular stimuli, such as LPS, can induce the phosphorylation and degradation of IκBα, and then, NF-κB is separated from IκBα and translocates into the nucleus, where it regulates the expression of pro-inflammatory cytokines, such as IL-6 and TNF-α. Therefore, we explored the effects of CQMUH-011 on LPS-induced NF-κB activation and IκBα degradation in primary microglia by Western blot analysis. As shown in Fig. 5b, phosphorylation levels of NF-κB p65 in the nuclear fraction were dramatically increased in LPS-stimulated microglial cells compared with those in cells without treatment. CQMUH-011 treatment markedly attenuated the nuclear translocation of NF-κB p-65. LPS also clearly accelerated IκBα phosphorylation, which also was significantly blocked by pretreatment with CQMUH-011.

### Effects of CQMUH-011 on the MAPK Signaling Pathways in LPS-Stimulated Microglia

**Fig. 6:**
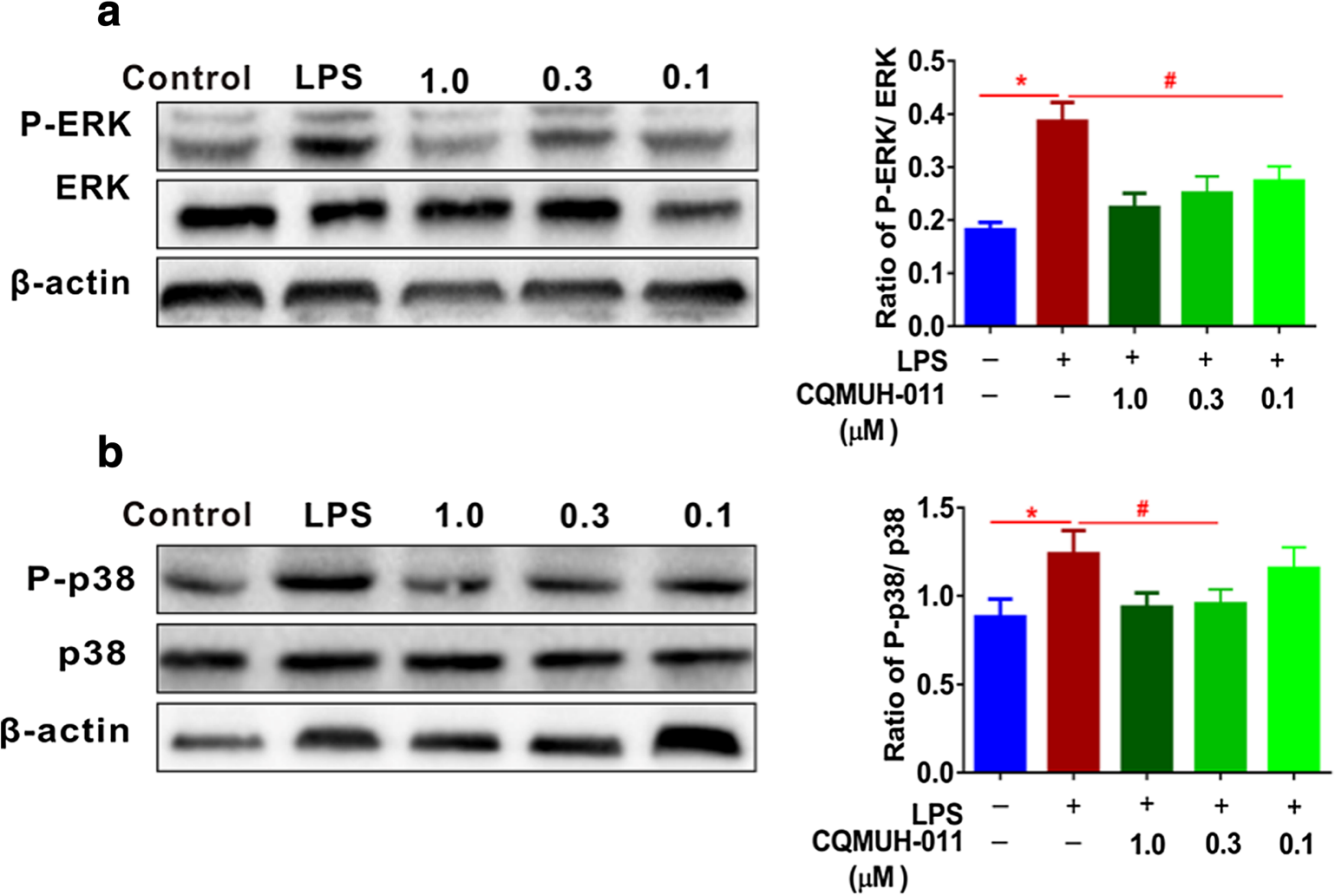
Inhibitory effects of CQMUH-011 on MAPK signaling. **a**, **b** Western blot analysis of MAPKs signaling proteins P-ERK, ERK, P-p38, and p38 expression in LPS-induced primary microglial cells. Histograms presenting relative ratios of phosphorylated to total ERK and p38 in different groups. Data are expressed as the means ± SD (*n* = 3 per group). *P < 0.05 LPS group *vs.* control group; ^#^*P* < 0.05 CQMUH-011 group *vs.* LPS group.

It is well-known that the phosphorylation of MAPKs plays an important role in the expression of inflammatory factors. We explored the effect of CQMUH-011 on the LPS-induced phosphorylation of MAPKs in primary microglial cells *via* Western blotting analyses. These results showed that phospho-p38 and phospho-ERK protein levels in LPS-induced primary microglial cells were significantly increased compared to those in LPS-untreated control cells. This finding suggests that MAPK signaling pathways are activated. Treatment with CQMUH-011 significantly decreased the expression of phospho-p38 and phospho-ERK proteins compared to the primary microglial cells stimulated with LPS in a concentration-dependent manner (Fig. 6a, b).

### CQMUH-011 Alleviates Focal Ischemia-Induced Brain Injury in Mice

**Fig. 7:**
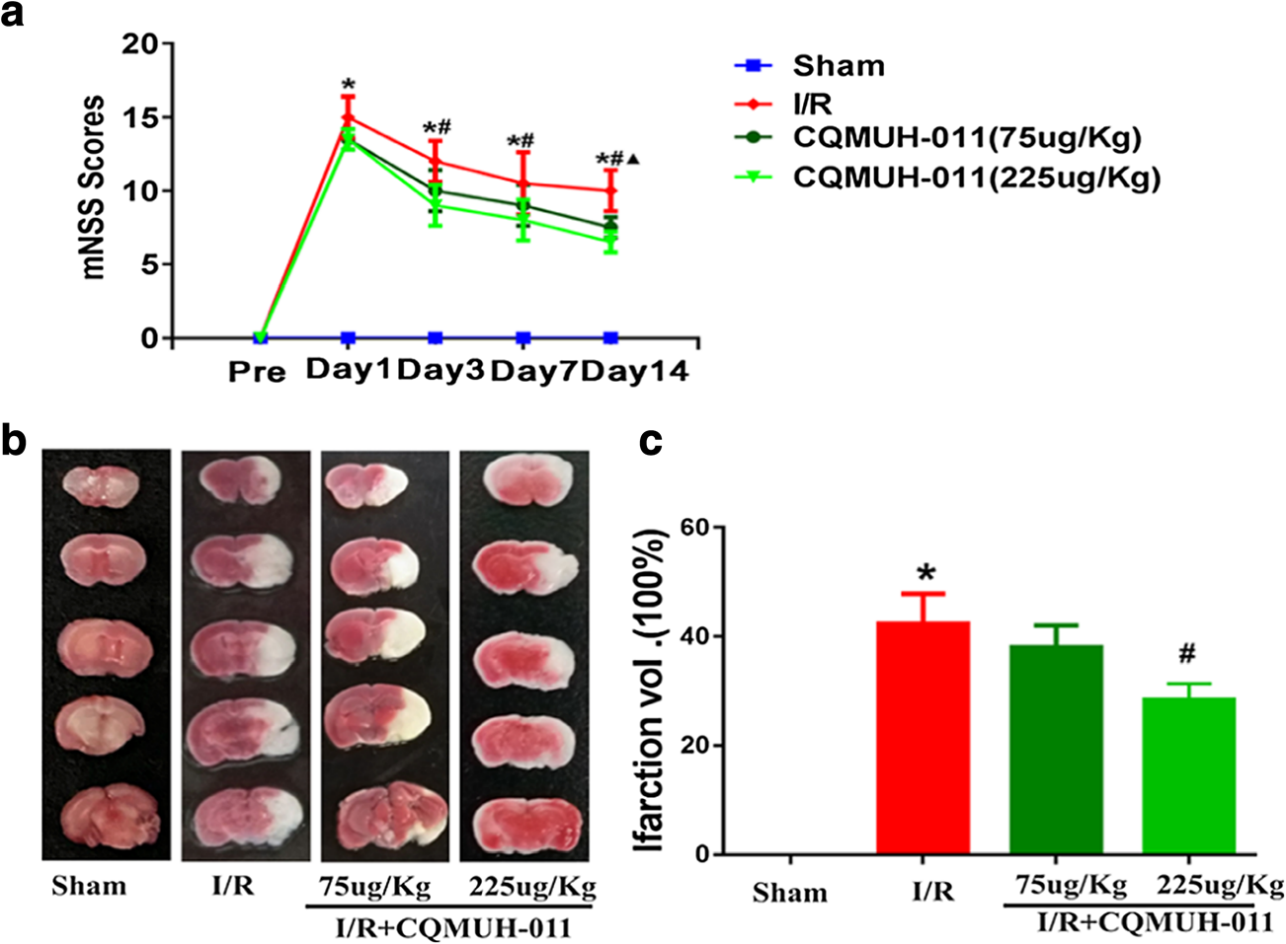
CQMUH-011 protects the brain against transient focal ischemia in mice. **a** Modified neurological severity score of mice subjected to I/R in the four groups (*n* = 8 per group, *P < 0.05 I/R *vs.* sham group, ^#^*P* < 0.05 CQMUH-011, 225 μg/kg *vs.* I/R group, ^▲^*P* < 0.05 CQMUH-011 75 μg/kg *vs.* I/R group). **b** Representative photographs of TTC-stained brain sections of different groups. **c** Infarct volumes of mice brain presented as a percentage of intact hemisphere. Data are expressed as mean ± SD (*n* = 3 per group; *P < 0.05 I/R *vs.* sham group, ^#^*P* < 0.05 CQMUH-011 *vs.* I/R group).

We examined whether CQMUH-011 protects the brain against ischemia induced by different time points of reperfusion after focal ischemia. As shown in Fig. 7a, the t-MCAO injury resulted in a dramatic increase in mNSS scores in comparison with the sham-operated group. While CQMUH-011 (225 μg/kg) treatment reduced the mNSS scores at 3, 7, and 14 days after t-MCAO, which suggests that CQMUH-011 treatment improves the functional outcome after cerebral I/R injury.

Additionally, consecutive brain sections were stained with TTC 1 day after t-MCAO. In comparison with the I/R group, CQMUH-011 (225 μg/kg) treatment of mice markedly decreased the volume of the infarct (*P* < 0.05; Fig. 7b, c). These results indicate that the CQMUH-011 treatment significantly reduces brain ischemic area caused by I/R injury.

### Effects of CQMUH-011 on Microglia Amount

**Fig. 8:**
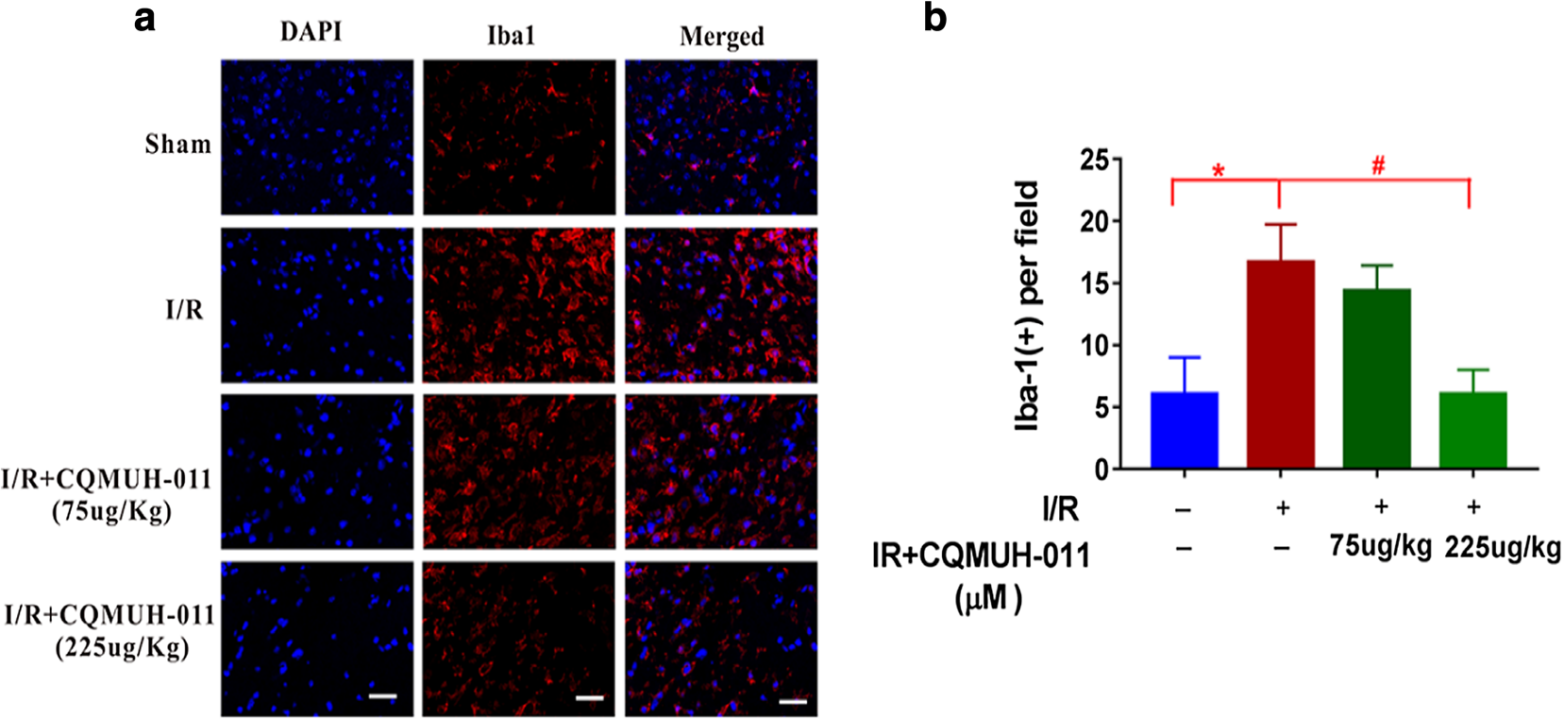
The Iba-1 immunofluorescent staining around the injury site in the brain sections of mice. The number of Iba-1-positive cells was expressed as mean ± SD. **a** Red staining in panels represent the Iba-1-positive staining. **b** The respective numbers of Iba-1-positive cells per field of mice (*n* = 5 per group; *P < 0.05 I/R *vs.* sham group, ^#^*P* < 0.05 CQMUH-011 *vs.* I/R group); scale bar = 50 um.

Because the inhibiting effect on inflammatory cytokines focused on day 3 post-t-MCAO, we only explored the effect of CQMUH-011 on microglia amount at this time point. The bright red staining was Iba-1-positive staining. Iba-1-positive staining was detected in the sham, I/R, and CQMUH-011 groups (Fig. 8). The Iba-1-positive cells around the injury site were significantly increased in the I/R group compared to the sham group. The Iba-1-positive cells were significantly less in the CQMUH-011 (225 μg/kg) group compared to the I/R group on day 3 post-t-MCAO.

## DISCUSSION

Activated microglia and their ability to drive neuroinflammation have been suggested to play key roles in the pathogenesis of several neurodegenerative disorders [6, 19–21]. Accumulating studies have demonstrated that blocking microglial activation may be neuroprotective [20, 21]. Previous studies have demonstrated that adamantane derivatives have been closely involved in various enzymes and exhibited a wide range of therapeutic activities through its beneficial influence including anti-inflammatory, anti-viral, anti-neurodegenerative diseases [22]. Particularly, when the adamantane derivatives contain two lipophilic centers, which usually were designed as anti-inflammatory drugs [23], James et al. [24] have reported that a novel series of adamantane derivatives could antagonize the purinergic P2X_7_ receptor, which is highly expressed on immune cells including microglia, and the activation of this receptor was closely associated with the expression of pro-inflammatory cytokines. In addition, memantine, another known adamantane derivative, has shown protective effects against streptozotocin-induced astrocyte activation by reduction of inflammatory cytokines, NF-κB translocation, glial fibrillary acidic protein, cyclooxygenase-2, TNF-α expression, and oxidative-nitrosative stress [25]. As one of the adamantine derivatives, CQMUH-011 exhibited a notable anti-inflammatory effect and protective effects on LPS/D-GalN-induced FHF in our previous study. In the present study, employing LPS-induced microglial activation, we further investigated the effects of CQMUH-011 on LPS-activated primary microglia. The results demonstrated that CQMUH-011 significantly inhibited microglial proliferation in a concentration-dependent manner and markedly reduced microglial activation by decreasing the level of well-known pro-inflammatory mediators, including TNF-α and IL-1β. Furthermore, we found that CQMUH-011 blocked the cell cycle and promoted apoptosis. In addition, CQMUH-011 also inhibited activation of the TLR4/NF-κB signaling pathway, which is associated with inflammation in LPS-stimulated microglia. Phosphorylation of MAPKs such as ERK and p38 kinases was also inhibited by CQMUH-011.

LPS, as a strong activator of microglia, has been the most widely used microglial activator in experimental models because it is well-established [26, 27]. During the process of neuroinflammation, a variety of neurotoxic and pro-inflammatory mediators released by activated microglia result in neuronal injury [28, 29]. The overexpression of TNF-α, which is a pro-inflammatory mediator, has been reported to promote neuroinflammation and neuronal cell death in an AD mouse model [30]. Additionally, the suppression of TNF-α markedly improved the survival rate of neurons and microglia co-cultures [31]. IL-1β is an important pro-inflammatory cytokine in the immune response [32]. Previous studies have revealed that overproduction of IL-1β by microglia has strong association with the cognitive functioning in the aging brain [33]. The present study demonstrated that CQMUH-011 markedly inhibited the production of TNF-α and IL-1β in a concentration-dependent manner.

The activation of microglia leads to the induction of proliferation. The migration of activated microglia tends towards the site of damage and upregulates the expression of various inflammatory factors [31, 34]. Proliferation and activation of microglia is a prominent feature of AD. The proliferation and activation of microglia can exacerbate tau pathology and promote the secretion of inflammatory factors which can injure neurons directly [35]. Previous studies have proven that the inhibition of microglial proliferation and the induction of cell apoptosis may play crucial roles in the pathogenesis of neurodegenerative disease [36, 37]. Notably, our previous study found that CQMUH-011 has an obvious inhibitory effect on LPS-induced RAW264.7 cell proliferation and markedly induces apoptosis [11]. In accordance with these findings, our results demonstrated that LPS considerably promoted the transition of microglia from the G1 phase to the S and G2 phases. CQMUH-011 arrested cells in the G1 phase and induced apoptosis, as depicted by annexin V-FITC flow cytometry, which may be caused by the inhibition of microglial proliferation.

Accumulating studies have demonstrated that multiple signaling transduction pathways such as MAPKs, PI3K/Akt, PPAR-γ, and NF-κB have major implications in the processes of neuroinflammation [20, 38]. Among these pathways, the activated NF-κB signaling pathway has been well-studied in response to the inflammatory activation of microglia. Briefly, NF-κB contains two DNA-binding subunits and one inhibitory subunit. The activation of NF-κB takes place in the cytoplasm and is mediated by IκB proteins. The phosphorylation of IκB results in the degradation of the inhibitory subunit, thus facilitating the nuclear import of the DNA-binding subunits and leading to the nuclear translocation of NF-κB. Then, activated NF-κB regulates the expression of various pro-inflammatory cytokines, such as iNOS, TNF-α, IL-6, and IL-1β [39, 40]. Therefore, we test the effect of CQMUH-011 on NF-κB activation and IκBα degradation in this study. The results illustrated that CQMUH-011 treatment significantly attenuated nuclear translocation of NF-κB p65 subunit and IκBα degradation.

In addition, to explore whether CQMUH-011 can affect the signaling pathways upstream of NF-κB, we investigated the effects of CQMUH-011 on the TLR4/MyD88 and MAPK pathways. TLR4, as a member of the family of pattern-recognition receptors, has been suggested to be closely implicated in the inflammatory process. LPS binds to TLR4 on the surface of microglial cells, which then recruit the MyD88 signaling complex; together, they induce the activation of MAPK and NF-κB signaling pathways, finally upregulating the expression of pro-inflammatory cytokines [18, 41]. A recent study revealed that CQMUH-011 downregulated the production of multiple pro-inflammatory cytokines by inhibiting the TLR4/NF-κB signaling pathway in an LPS/D-GalN-induced fulminant hepatic failure model [11]. Therefore, this study hypothesized that CQMUH-011 may have an analogous effect on LPS-stimulated microglial cells. Thus, we evaluated the expression of phospho-NF-κB p65 and TLR4 in LPS-stimulated microglial cells by Western blotting and flow cytometry. The result confirmed the aforementioned hypothesis and demonstrated that CQMUH-011 significantly suppressed NF-κB activation and inhibited the protein expression of TLR4.

Furthermore, we investigated the effects of CQMUH-011 on the MAPK signaling pathway upstream of NF-κB. The MAPK signaling pathways take part in various pathological and physiological processes such as apoptosis, necroptosis, and proliferation [42, 43], which play crucial roles in inflammatory diseases [44, 45]. Previous studies have proven that inhibiting the activation of MAPK signaling pathways significantly attenuates the inflammatory response in microglial cells. This study confirmed that the phosphorylation of p38 and ERK was increased by treatment with LPS, but their phosphorylation was clearly inhibited by CQMUH-011. Thus, the above results demonstrate that the anti-neuroinflammatory effect of CQMUH-011 may be mediated *via* suppression of the NF-κB and MAPK signaling pathways.

The pathogenesis of cerebral I/R injury involves diverse mechanisms, but emerging studies have focused on the inflammatory response driven by microglia activation in its pathogenic progression [46–48]. Therefore, inhibition of excessive microglial activation-induced neuroinflammation may improve outcomes after cerebral I/R injury. The present study tested the possibility that CQMUH-011 can ameliorate neuroinflammation and inhibit microglial activation after t-MCAO. This analysis indicated that CQMUH-011 (225 μg/kg) treatment significantly improved the tissue and functional outcomes, reducing the amount of microglia around the injured site. Actually, few agents that target microglial activation in the context of stroke have reached clinical trials. This finding suggested that CQMUH-011 may be considered a potential therapeutic agent for the treatment of ischemic stroke.

## CONCLUSIONS

The present study demonstrated that CQMUH-011 significantly prevents microglial activation and its associated neuroinflammation by downregulating the production of various pro-inflammatory cytokines and mediators. These effects may be due to the inhibition of the TLR4/NF-κB and MAPK signaling pathways. Additionally, CQMUH-011 alleviates the neurological deficits and improves the histological outcomes associated with transient focal cerebral ischemia in mice. In view of the current findings, the novel adamantane sulfonamide compound CQMUH-011 may be a potent anti-neuroinflammatory agent for cerebral ischemia patients accompanied by microglial activation. However, the effect of CQMUH-011 on microglial phenotype polarization is still unclear and further studies will be included in our future work.

## Data Availability

All authors have confirmed that all data and materials support their published claims and comply with field standards.
